# A Defence of the Medical Profession of the United States

**Published:** 1846-04

**Authors:** 


*A defence of the Medical Profession of the United States*: being a Valedictory Address to the graduating class at the Medical Commencement of the University of New York. Delivered March 11, 1846, by Martyn Paine, A. M., M. D., Professor of the Institutes of Medicine and Materia Medica in the University of New York. &c. New York. 1846. pp. 24. (From the Author.)

The great enemy against whom Professor Paine volunteers to defend the Medical profession, is Dr. J. N. Davis, in connection with the New York State Medical Society. The former, who is styled "a young man in the township of Binghampton," having originated the plan for a National Convention, adopted by the latter, in which Professor Paine thinks he has discovered a jealousy of the existing medical schools. He particularly objects to the suggestion which has been made, of the propriety of increasing the term of instruction in our Medical Colleges. "It is oppression towards the poor for the sake of crippling our Medical Colleges," and again, "there is an *aristocratic* feature in this movement of the worst omen, however the spirit by which it is prompted may belong to the agrarian policy." The Professor is of opinion that the medical profession is pretty well off in this country, much better in regard to its "practical habits" and "successful treatment of disease," than any other country whatever, although he admits that "such evils" (as those complained of) "exist, * * and I also agree as to their becoming appropriate subjects for united deliberation."

Whether such a spirit as that ascribed to the State Medical Society actually exists, we have no means of knowing, but should not be at all surprised to find it were so. Not having means of competing on equal terms for the chairs in medical schools, it seems to us quite natural that the "young man in the township of Binghamton," and other young men in different townships, should think that there were some improvements still to be made in a country where each citizen is entitled to equal rights and privileges. On the other hand it is not surprising that those who hold high and profitable situations in the medical schools, should see little to be done in the way of improvement.

Let there be established a system of *concours* or public trials, where all may enjoy equal advantages in competing for valuable situations, and jealousies of medical schools will cease to exist. Holding the interests of the great body of the profession above that of schools or societies, (if they conflict,) thinking also, that however advanced we may be, *progress* should still be our watchword, we have desired to see assembled a body like that contemplated, composed of members from every part of the country. Whether its action will be beneficial or otherwise must be seen and judged of by the medical public at the proper time.

In the mean time, without feeling any direct interest in the controversy likely to ensue, we think the author of this address assumes a position which he is not entitled exclusively to occupy, for we are happy to know that there are many even in the far west who are not surpassed in zeal for the true "honor and dignity of the profession." "And now, *again*, gentlemen, for the third time, I stand up *alone* in the broad expanse of America, in an open defence of the honor and dignity of the profession."

